# NovaBone Bioactive Glass for Oral Bone Regeneration: Mechanisms, Innovations, and Clinical Outcomes

**DOI:** 10.7759/cureus.108789

**Published:** 2026-05-13

**Authors:** Mohamed Amir Khan, Asad Mujawar, Akriti Agrawal, Debi P Acharya, Deepti Krishnan Kutty, Laxman Roy Chittaluri, Kafeel Ahmed, Rubeena Naaz

**Affiliations:** 1 Department of Oral and Maxillofacial Surgery, King Khalid University, Buraydah, SAU; 2 Department of Prosthodontics and Implantology, Mazaya Dental Center, Muhayil, SAU; 3 Department of Dentistry, Veer Surendra Sai Institute of Medical Sciences and Research, Sambalpur, IND; 4 Department of Oral and Maxillofacial Surgery, Dental College Azamgarh, Azamgarh, IND; 5 Department of Oral and Maxillofacial Surgery, Kunhitharuvai Memorial Charitable Trust (KMCT) Dental College, Calicut, IND; 6 Department of Oral and Maxillofacial Surgery, Mamata Dental College and Hospital, Khammam, IND; 7 Department of Periodontology, MNR Dental College and Hospital, Sangareddy, IND; 8 Department of Dentistry, SRK Dental Clinic, Hyderabad, IND

**Keywords:** bioactive glass, calcium phosphosilicate, implant dentistry, novabone putty, oral bone regeneration, periodontal regeneration, socket preservation

## Abstract

Bone regeneration remains a fundamental component of contemporary oral and implant dentistry, particularly in the management of post-extraction defects, periodontal bone loss, and implant site deficiencies. Among available grafting materials, synthetic bioactive substitutes have gained attention due to their ability to combine scaffold function with biologically active surface properties. NovaBone, a calcium phosphosilicate-based bioactive glass, has emerged as a clinically relevant option owing to its capacity for ionic dissolution, surface reactivity, and the promotion of mineralized tissue formation. This review aims to provide a comprehensive synthesis of the mechanistic basis, material characteristics, and clinical performance of NovaBone in oral bone regeneration. The biological activity of NovaBone is primarily driven by the release of silicon (Si), calcium (Ca), and phosphate ions, which contribute to osteogenic signaling, angiogenesis, and the formation of a hydroxycarbonate apatite (HCA) layer that facilitates bonding with host bone. In addition to its physicochemical properties, advancements in formulation, including putty and composite systems, have improved its clinical handling and adaptability. Clinical evidence indicates that NovaBone demonstrates favorable outcomes in periodontal intrabony and furcation defects, with additional applicability in socket preservation, ridge augmentation, sinus elevation, and peri-implant defect reconstruction. However, the available literature shows variability in study design, follow-up duration, and outcome measures, with relatively limited long-term randomized data specific to NovaBone formulations. Overall, NovaBone represents a promising bioactive graft material that integrates scaffold function with biologically driven regeneration. Its clinical utility is best understood within a mechanism-oriented framework that links material behavior to tissue response. Future research should focus on standardized clinical protocols and high-quality comparative trials to further define its role among contemporary regenerative biomaterials in implant dentistry.

## Introduction and background

Oral bone loss remains a major challenge in contemporary implant dentistry because the alveolar process is highly dependent on the presence of teeth for structural maintenance. Following extraction, physiological remodeling leads to a marked dimensional reduction of the ridge, with horizontal loss generally exceeding vertical loss during early healing. In a widely cited systematic review of human studies, post-extraction sites showed substantial hard tissue contraction within the first three to six months, emphasizing why untreated socket healing can compromise later implant positioning and prosthetically driven rehabilitation [1]. Beyond extraction-related remodeling, hard tissue deficiencies relevant to implant therapy may also arise from periodontal destruction, peri-implant disease, trauma, and other local pathologic processes, all of which can reduce the volume and architecture required for stable implant placement [2,3].

Adequate bone volume is a fundamental prerequisite for predictable implant-supported rehabilitation. Successful implant placement depends not only on osseointegration at the implant surface but also on correct three-dimensional (3D) positioning within a sufficient osseous envelope. When residual ridge dimensions are inadequate, regenerative or augmentation procedures are often required before or during implant therapy to improve both functional loading conditions and esthetic outcomes [3,4]. For this reason, bone regeneration is not merely an adjunctive procedure in implant dentistry; in many partially or fully edentulous situations, it is central to treatment planning and long-term restorative success [3,4].

A wide range of grafting materials has therefore been employed in oral reconstruction, including autografts, allografts, xenografts, and synthetic substitutes. Although autogenous bone has long been regarded as a reference material because of its biological compatibility and regenerative potential, its use is constrained by limited availability, donor-site morbidity, increased operative burden, and variable resorption behavior [5,6]. Allografts and xenografts broaden therapeutic options, but they are also associated with important limitations, including processing-related variability, slower remodeling, and concerns related to biological safety, immunologic response, or patient acceptance, depending on the source and indication [5,6]. These constraints have driven sustained interest in synthetic alternatives that can provide defect fill, scaffold function, and biologically favorable healing without the disadvantages of donor harvesting [5,6].

Among synthetic graft substitutes, bioactive glass-based materials have attracted particular attention because they offer more than passive space maintenance. Unlike inert fillers, bioactive glasses interact dynamically with the host environment through surface reactions and controlled ionic dissolution. Their regenerative effect is linked to the release of ions that can modulate cellular behavior relevant to osteogenesis and angiogenesis, while their reactive surface supports the formation of a hydroxycarbonate apatite (HCA) layer that promotes bonding with surrounding hard tissues [7,8]. In dentistry, these properties have supported the use of bioactive glass in periodontal regeneration, ridge preservation, defect grafting, and other craniofacial applications, making it one of the most clinically relevant classes of alloplastic materials in oral regeneration research [8].

NovaBone is a commercially used bioactive glass graft material based on calcium phosphosilicate chemistry and is available in moldable putty formulations designed for clinical handling in bony defects [8-10]. Its appeal in oral regeneration lies in the combination of scaffold support, surface bioactivity, and a biologically active dissolution profile that distinguishes it from many conventional alloplasts [7,8]. Experimental and clinical studies have reported favorable regenerative responses with NovaBone-containing formulations in intrabony periodontal defects and other osseous indications, including improvements in clinical attachment parameters, probing depth reduction, radiographic defect fill, and histologic evidence of new bone formation in preclinical models [9-12]. At the same time, the available literature remains heterogeneous in design, indication, comparator material, and outcome reporting, which makes a focused synthesis clinically valuable.

Clinical context

Oral bone loss commonly occurs after tooth extraction, periodontal disease, trauma, infection, or peri-implant defects, often compromising implant placement and long-term prosthetic stability. Bone grafting is therefore frequently required to preserve or rebuild alveolar bone volume. While autografts, allografts, and xenografts are commonly used, they may be associated with limitations such as donor-site morbidity, variable availability, disease-transmission concerns, or inconsistent resorption. These challenges have increased interest in synthetic graft materials such as NovaBone.

Literature gap

Although bioactive glass materials have been widely investigated for bone regeneration, the evidence specifically focusing on NovaBone in oral and implant-related applications remains scattered across different clinical indications, defect types, and study designs. There is a need for a focused review that summarizes its biological mechanisms, clinical applications, advantages, and current limitations in oral bone regeneration.

Accordingly, the aim of this review is to synthesize the mechanistic basis, material innovations, and clinical outcomes associated with NovaBone bioactive glass in oral bone regeneration. By integrating foundational biomaterial science with translational and clinical evidence, this review seeks to clarify where NovaBone currently stands within regenerative implant dentistry and where future evidence is still needed [7-12].

## Review

Method

This narrative review was conducted to synthesize the available literature on NovaBone and related calcium phosphosilicate bioactive glass materials in oral bone regeneration. This review did not include a formal systematic search strategy, meta-analysis, or meta-regression. Relevant peer-reviewed publications were selected from major scientific databases and reference lists, with an emphasis on studies related to bioactive glass, NovaBone, oral bone regeneration, periodontal defects, and implant-related applications.

Publications were considered for inclusion if they addressed one or more of the following domains: biomaterial composition and physicochemical properties, biological mechanisms of action, formulation and technological innovations, and clinical applications or outcomes in oral and implant dentistry. Priority was given to studies evaluating NovaBone directly; however, the broader bioactive glass literature was also included where NovaBone-specific evidence was limited, particularly to provide a mechanistic and translational context. The selected literature included experimental studies, clinical studies, randomized trials, histologic and histomorphometric investigations, and relevant review articles. The evidence was organized narratively into sections covering composition and properties, biological mechanisms, technological innovations, clinical applications, and evidence synthesis.

Composition and material properties of NovaBone

NovaBone is a synthetic bioactive graft material primarily composed of calcium phosphosilicate, a class of silica-based bioactive glass specifically engineered for bone regeneration applications. This composition is derived from the broader family of bioactive glasses, which typically include a network of silicon dioxide (SiO₂) combined with calcium oxide (CaO) and phosphorus pentoxide (P₂O₅), forming a reactive glass matrix capable of interacting with biological fluids through a sequence of physicochemical reactions. Upon contact with body fluids, the material undergoes ion exchange and a partial dissolution of the silica network, resulting in the release of calcium, phosphate, and silicon ions. This is followed by the formation of a silica-rich surface layer and the subsequent precipitation of a calcium phosphate layer, which crystallizes into hydroxycarbonate apatite (HCA). The HCA layer closely resembles the mineral phase of natural bone, facilitates bonding with host bone, and supports osteogenic activity during bone regeneration [13,14].

The calcium phosphosilicate structure of NovaBone is characterized by a disordered, amorphous network, which facilitates rapid surface reactions upon implantation. Unlike crystalline biomaterials, this amorphous arrangement allows controlled degradation and ion exchange, which is essential for its regenerative function [13].

A key feature of NovaBone is its ionic dissolution profile, which involves the release of biologically active ions such as calcium (Ca²⁺), phosphate (PO₄³⁻), and silicon (Si⁴⁺) when exposed to physiological fluids. These ions are released in a controlled manner and contribute directly to cellular signaling pathways involved in osteogenesis and angiogenesis [15,16]. Studies have demonstrated that silicon ions play an important role in stimulating osteoblast differentiation; however, this effect is concentration-dependent, with moderate silicon concentrations generally supporting osteogenic activity while excessive levels may impair cell viability. Calcium and phosphate ions further contribute to mineral deposition and bone matrix formation [15]. The synergistic effect of these ionic products enhances cellular proliferation and supports the early stages of bone regeneration [16].

Physicochemical Characteristics

The regenerative performance of NovaBone is largely governed by its surface reactivity, which is a defining characteristic of bioactive glass materials. Upon implantation, the material undergoes a sequence of physicochemical reactions beginning with ion exchange and silica network dissolution. This leads to the formation of a silica-rich gel layer on the surface, followed by the precipitation of an amorphous calcium phosphate layer that subsequently crystallizes into HCA [17].

The formation of the HCA layer is particularly significant because it closely resembles the mineral phase of natural bone, enabling direct bonding between the graft material and host tissue. This property distinguishes bioactive glass from many conventional graft materials that rely primarily on passive integration rather than active chemical bonding [17,18].

In addition to surface reactivity, NovaBone exhibits favorable porosity and scaffold characteristics. The material provides a three-dimensional framework that supports cell attachment, migration, and vascular ingrowth. Although its porosity is generally lower than that of some natural grafts, the interconnected microstructure is sufficient to facilitate nutrient diffusion and tissue infiltration, thereby promoting new bone formation [18].

From a clinical standpoint, NovaBone is available in various handling forms, including particulate granules and moldable putty formulations. The putty form is particularly advantageous in oral and maxillofacial applications, as it improves handling, adaptability to defect morphology, and stability within the surgical site without the need for additional containment systems [19].

Bioactivity and Osteoconductivity

NovaBone demonstrates both bioactivity and osteoconductivity, which are critical for effective bone regeneration. Bioactivity refers to the material's ability to elicit a biological response through surface reactions, while osteoconductivity describes its capacity to serve as a scaffold for new bone growth.

Compared to traditional graft materials such as hydroxyapatite or β-tricalcium phosphate, bioactive glass exhibits a more dynamic interaction with the host environment. Conventional materials are largely passive and depend on host-mediated remodeling, whereas bioactive glass actively participates in regeneration through ionic signaling and surface transformation [18,20].

One of the most important features of NovaBone is its ability to form a chemical bond with the bone via the HCA layer, enabling stable integration at the graft-bone interface. This surface-mediated bonding enhances the mechanical stability of the regenerated site and supports long-term implant success [17,20]. Furthermore, the continuous release of therapeutic ions promotes osteoblastic activity and angiogenesis, thereby accelerating the healing process compared to inert graft materials (Table 1) [15,20].

**Table 1:** Composition and properties of NovaBone versus other graft materials HCA: hydroxycarbonate apatite

Property	NovaBone (bioactive glass)	Hydroxyapatite	β-Tricalcium phosphate	Xenograft
Primary composition	Calcium phosphosilicate-based bioactive glass [13,14]	Synthetic calcium phosphate [20]	Synthetic calcium phosphate ceramic [20]	Deproteinized bone of animal origin [18]
Structural nature	Amorphous glass network [13,14]	Crystalline [20]	Crystalline [20]	Natural mineral scaffold with processed organic removal [18]
Ionic release profile	Releases Ca²⁺, PO₄³⁻, and Si⁴⁺ ions that influence bone healing [15,20]	Minimal ionic release compared to bioactive glass [20]	Moderate calcium and phosphate release during resorption [20]	Limited biologically active ionic release [18]
Surface reactivity	High surface reactivity with rapid biological interaction in body fluids [17,20]	Relatively low surface reactivity [20]	Moderate reactivity [20]	Primarily passive scaffold behavior [18]
Hydroxycarbonate apatite layer formation	Forms an HCA layer that supports direct interfacial bonding to the bone [17]	Does not characteristically form a bioactive glass-type HCA surface reaction layer [20]	Does not characteristically form a bioactive glass-type HCA surface reaction layer [20]	No intrinsic HCA-forming bioactive surface reaction [18]
Bioactivity	High bioactivity [13,17]	Lower than bioactive glass [20]	Moderate [20]	Limited intrinsic bioactivity [18]
Osteoconductivity	Osteoconductive scaffold with additional ionic stimulation [15,20]	Osteoconductive [20]	Osteoconductive [20]	Osteoconductive [18]
Osteostimulatory potential	Present through ionic dissolution products and cellular signaling effects [15]	Generally absent [20]	Limited [20]	Generally absent [18]
Bone-bonding mechanism	Surface-mediated chemical bonding through HCA layer formation [17]	Mainly physical apposition and scaffold support [20]	Mainly scaffold-guided bone ingrowth [20]	Primarily scaffold-mediated integration [18]
Porosity/scaffold behavior	Supports cell attachment and tissue ingrowth; functions as a regenerative scaffold [18,20]	Porous scaffold depending on formulation [20]	Porous resorbable scaffold [20]	Natural porous architecture favorable for ingrowth [18]
Resorption behavior	Controlled degradation with concurrent ionic release [14,20]	Very slow resorption [20]	Faster resorption than hydroxyapatite [20]	Slow remodeling and resorption [18]
Handling forms	Available as particles and putty for improved defect adaptation [19]	Commonly granules or blocks [20]	Commonly granules or blocks [20]	Commonly granules or blocks [18]
Clinical handling advantage	Moldable putty form improves placement and containment in irregular defects [19]	May require additional stabilization depending on defect morphology [20]	May require defect containment [20]	Often requires membrane/support depending on the site [18]

Compared to conventional graft substitutes, NovaBone demonstrates a distinct combination of surface bioactivity, ionic release, and HCA-mediated bone bonding, which may contribute to its regenerative potential beyond passive osteoconduction alone [15,17,20].

Biological mechanisms of bone regeneration

Ionic Dissolution and Cellular Signaling

A central feature of NovaBone and related bioactive glass systems is their ability to undergo controlled ionic dissolution after contact with physiological fluids. This reaction releases soluble ionic species, particularly silicon, calcium, and phosphate, which do not act merely as degradation by-products but function as biologically relevant signals within the healing environment [21-24]. Experimental work has shown that soluble silica can enhance osteogenic differentiation and matrix mineralization, while calcium-rich dissolution products support early mineral deposition and osteoblast activity [22,23]. In osteoblastic cells, silica-based bioactive glasses have also been shown to stimulate the expression of bone-related genes, including growth factors and matrix-associated transcripts, indicating that the material can influence regeneration at the molecular level rather than serving only as a passive scaffold [23,24].

This ionic signaling is important because bone regeneration requires the coordinated activation of proliferation, differentiation, extracellular matrix production, and mineralization. In vitro studies have reported that conditioned media derived from bioactive glass can increase the expression of osteogenic markers such as alkaline phosphatase, osteopontin, osteocalcin, and bone morphogenetic protein-related pathways, thereby supporting the maturation of osteoprogenitor and osteoblast-lineage cells [23-26]. The osteogenic response appears to depend not only on the presence of silicon and calcium ions but also on their concentration, release kinetics, and the overall composition of the glass network [21,22,25].

Osteostimulation and Osteoconduction

Beyond its scaffold role, bioactive glass exhibits osteostimulatory behavior, meaning that it can actively promote a pro-regenerative cellular phenotype. This distinguishes it from materials that are only osteoconductive. In cell-based and scaffold-based studies, bioactive glass has been associated with improved osteoblast adhesion, proliferation, differentiation, and matrix formation, supporting the concept that the material contributes to bone healing through both structural and biological mechanisms [24-27]. The term "osteostimulation" is therefore appropriate in the context of bioactive glass because regenerative effects are mediated not only by defect filling and space maintenance but also by the ionic and surface-driven stimulation of bone-forming cells [21,24].

Angiogenesis is another critical component of successful regeneration, as new blood vessel formation is essential for oxygen delivery, nutrient exchange, and the coupling of bone formation with tissue remodeling. Reviews and experimental studies have shown that bioactive glass compositions can promote angiogenic responses through the stimulation of endothelial behavior and the upregulation of angiogenesis-related mediators, including vascular endothelial growth factor-associated pathways [27,28]. This dual effect on osteogenesis and angiogenesis is especially relevant in oral bone regeneration, where graft performance depends on rapid biological integration in a confined and mechanically demanding environment [27,28].

Formation of the HCA Layer

One of the defining mechanisms underlying the clinical performance of bioactive glass is the formation of a surface HCA layer after implantation. When exposed to body fluids, the glass surface undergoes ion exchange, silica network dissolution, reprecipitation of a silica-rich layer, and subsequent nucleation of a calcium phosphate phase that matures into a carbonated apatite layer similar to the mineral phase of the bone [29,30]. This surface transformation is fundamental to the concept of bioactivity because it creates a biologically recognizable interface that supports strong integration with host tissue [29].

The HCA layer contributes to interface bonding with the surrounding bone and favors biomimetic mineralization at the graft surface. Studies examining mineralization behavior and interfacial properties have shown that apatite formation enhances conjunction performance and provides a more favorable substrate for tissue bonding and matrix deposition [30]. In practical terms, this means that NovaBone does not simply occupy space within a defect; rather, it evolves into a chemically active interface that supports bone apposition and biological fixation [29,30].

Immunomodulatory Effects

The early inflammatory phase strongly influences the eventual success of bone regeneration, and increasing evidence shows that bioactive glass can modulate this phase in a favorable direction. Macrophages are among the first cells to interact with implanted biomaterials, and their polarization profile can shape whether healing proceeds toward chronic inflammation or constructive regeneration [31]. Studies on bioactive glass-containing systems indicate that these materials can influence macrophage behavior, reducing excessive pro-inflammatory signaling while promoting a more regenerative microenvironment [31,32].

This immunomodulatory effect is important because bone healing is now recognized as an osteoimmune process rather than a purely skeletal one. Bioactive glass has been reported to alter cytokine responses and macrophage polarization in ways that may support tissue repair, angiogenesis, and subsequent osteogenesis [31,32]. Accordingly, the regenerative potential of NovaBone may be understood not only through osteoblast-centered mechanisms but also through its capacity to shape the inflammatory niche in which bone formation occurs [31,32].

**Figure 1:**
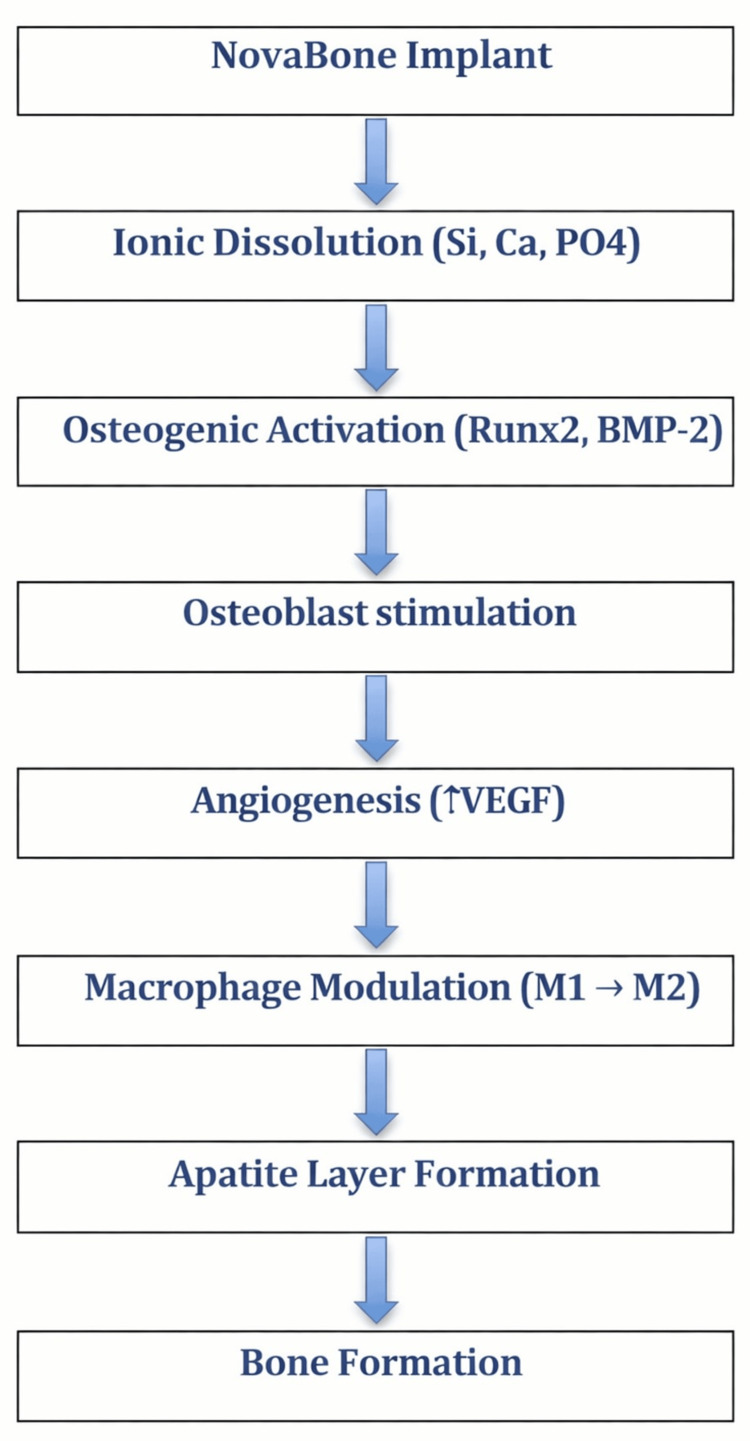
Proposed mechanism of action of NovaBone in oral bone regeneration Generated by the authors using Microsoft Word (Microsoft Corp., Redmond, WA) VEGF: vascular endothelial growth factor

After implantation, calcium phosphosilicate undergoes ionic dissolution with the release of silicon (Si), calcium (Ca), and phosphate ions, leading to osteogenic gene activation, osteoblast stimulation, angiogenic signaling, macrophage modulation, and the formation of an HCA layer that promotes bone bonding and new bone formation (Figure 1) [21-32].

Innovations in NovaBone technology

Advanced Formulations

One major area of innovation in bioactive glass technology has been the refinement of handling characteristics without losing biological performance. Traditional particulate grafts offer defect fill but may be difficult to stabilize in irregular or non-contained defects. In response, putty-based and injectable formulations have been developed to improve adaptability, surgical convenience, and defect conformity [33,34]. For NovaBone specifically, moldable putty formulations have been clinically attractive because they permit easier packing and better adaptation to defect morphology than loose particles alone, which is particularly useful in oral defects with limited containment [33].

Injectable systems represent a further development of this concept. Reviews of injectable bioactive glass-based composites indicate that combining bioactive glass with hydrogel or polymeric carriers can improve placement, minimally invasive delivery, and retention at the target site while preserving regenerative signaling potential [34]. These approaches are important because clinical efficacy is influenced not only by chemistry but also by how reliably the material can be delivered and maintained within the defect during early healing [33,34].

Composite Approaches

Another important innovation has been the combination of bioactive glass with autologous biologics and polymer-based matrices. Among these, platelet-rich fibrin (PRF) has received particular attention because it can supply fibrin architecture, growth factors, and handling synergy when mixed with graft particles or putty [35-38]. Clinical studies on intrabony periodontal defects have shown that the addition of PRF to bioactive glass can improve clinical and radiographic outcomes compared to grafting alone, although the degree of additional benefit varies by study design and defect characteristics [35-37]. These composite strategies are attractive because they aim to integrate scaffold function, ionic bioactivity, and biological signaling within a single regenerative construct [35-38].

Hybrid scaffolds combining bioactive glass with natural or synthetic polymers have also expanded the functional range of these materials. Reviews on bioactive glass/polymer composites describe improvements in toughness, handling, and regenerative versatility while still retaining the ability to support osteogenesis and angiogenesis [38,39]. Such systems may be particularly relevant for future oral regenerative applications where defect-specific mechanics and controlled release are needed, alongside conventional grafting performance [38,39].

Surface and Structural Modifications

The structural modification of bioactive glass has led to the development of nanostructured and mesoporous forms with higher surface area, more controlled dissolution, and improved capacity for molecule loading. Mesoporous bioactive glasses are especially important because their ordered pore systems and high specific surface area can intensify surface reactivity, enhance apatite formation, and permit the delivery of ions, drugs, or osteogenic agents [40-43]. Compared to conventional melt-derived glasses, these nanostructured variants offer more opportunities for tailoring biological behavior through pore design and composition [40-42].

Enhanced porosity is another major innovation. Hierarchical porous architectures can improve cell migration, nutrient diffusion, vascular infiltration, and bone ingrowth, all of which are essential in three-dimensional tissue regeneration [41,43,44]. Reviews of bioactive glass scaffolds emphasize that multiscale porosity, spanning mesopores to macropores, may better replicate the requirements of living bone than dense or minimally porous substitutes [41,43]. These developments are highly relevant for future generations of NovaBone-like materials, even if not all such modifications are yet standard in currently marketed formulations [40-44].

Emerging Research Trends

Current research increasingly positions bioactive glass within broader tissue-engineering strategies rather than as a stand-alone graft substitute. One major direction is integration with cell-instructive scaffolds and stem-cell-compatible matrices for defect-specific regeneration [40,42,45]. Another is the development of 3D-printed and architecturally controlled scaffolds capable of combining bioactive ion release, structural support, and spatially defined porosity [44,45]. These platforms are particularly promising for craniofacial and oral applications where defect geometry is often complex and where the personalization of scaffold design could improve reconstruction outcomes [44,45].

In parallel, multifunctional systems are being explored in which bioactive glass acts as both a regenerative phase and a delivery platform for therapeutic ions, drugs, peptides, or biologically active coatings [42,46]. Taken together, these trends suggest that the future of NovaBone-related technology is likely to move toward more customizable, hybrid, and biologically integrated systems that combine handling convenience with enhanced regenerative precision (Table 2) [34,40,42,46].

**Table 2:** Recent innovations and modifications in bioactive glass systems PRF, platelet-rich fibrin; 3D, three-dimensional

Innovation area	Key concept	Relevance to NovaBone-oriented regeneration	Supporting references
Putty formulations	A moldable carrier improves adaptation and stability	Better handling of irregular oral defects	[33]
Injectable systems	Bioactive glass combined with hydrogel/polymer carriers	Minimally invasive placement and improved defect retention	[34]
PRF/biological composites	Addition of autologous fibrin and growth factors	Potential enhancement of soft-tissue healing and bone fill	[35-38]
Polymer-bioactive glass hybrids	Combines toughness and biological activity	Improved handling and multifunctionality	[38,39]
Mesoporous bioactive glass	High surface area and ordered pore channels	Faster surface reactions and a potential delivery platform	[40-42]
Hierarchical porosity designs	Multiscale pores support vascular and tissue ingrowth	Better scaffold performance in 3D regeneration	[41,43,44]
3D-printed scaffolds	Architecture can be tailored to the defect geometry	Potential for personalized craniofacial regeneration	[44,45]
Multifunctional systems	Combine ion release with the delivery of drugs or therapeutic agents	Expands regenerative and anti-infective potential	[42,46]

Clinical applications in oral bone regeneration

Socket Preservation

Socket preservation is intended to reduce the dimensional changes that occur after tooth extraction and to maintain a ridge contour more suitable for later implant placement. Bioactive glass-based grafts have shown usefulness in limiting post-extraction alveolar resorption and supporting mineralized tissue formation during socket healing [47,48]. Histomorphometric studies of calcium phosphosilicate putty in extraction sockets have also demonstrated new bone formation before implant placement, supporting its relevance to NovaBone-type materials [49,50].

Ridge Augmentation

In implant site development, calcium phosphosilicate putty has been used for reconstructive grafting and ridge augmentation because it adapts well to defects, supports clot stability, and serves as a bioactive scaffold during healing [51]. Clinical series have reported the favorable implant-related use of this material in grafted sites, suggesting that NovaBone can contribute to ridge reconstruction, especially where a moldable synthetic graft is preferred [51].

Role in Implant Site Development and Osseointegration

NovaBone may support osseointegration through its bioactive surface reactions. After implantation, calcium phosphosilicate bioactive glass releases calcium, phosphate, and silicon ions, which contribute to the formation of an HCA layer on the graft surface [7,13,14,17]. This apatite layer is chemically similar to the mineral phase of the bone and may encourage direct bone bonding [13,17,20]. In addition, ionic dissolution products from bioactive glass have been shown to stimulate osteoblast activity, osteogenic differentiation, and mineralized tissue formation, thereby creating a favorable environment for bone-to-implant contact during healing [7,15,21-24]. Clinical studies using calcium phosphosilicate putty in extraction sockets and implant-related sites further support its potential role in implant site development and osseointegration [49-51].

Sinus Lift Procedures

Bioactive glass has also been studied in maxillary sinus augmentation. Clinical and histologic studies have shown that bioactive glass used alone or with autogenous bone can support sinus floor elevation, mineralized tissue formation, and subsequent implant placement [52-55]. These findings support the clinical relevance of calcium phosphosilicate technology in sinus grafting, although not every study specifically used the NovaBone commercial formulation [52-55].

Periodontal Defect Regeneration

Periodontal regeneration is the indication with the strongest direct NovaBone evidence. Clinical studies of NovaBone putty in intrabony defects have reported probing depth reduction, clinical attachment gain, and radiographic defect fill [56-59]. Comparative and adjunctive studies have further shown that NovaBone used with platelet concentrates may improve regenerative outcomes in selected intrabony and vertical periodontal defects [57,58]. Furcation studies also support the usefulness of NovaBone putty in grade II furcation lesions [60].

Peri-implant Defects

**Figure 2:**
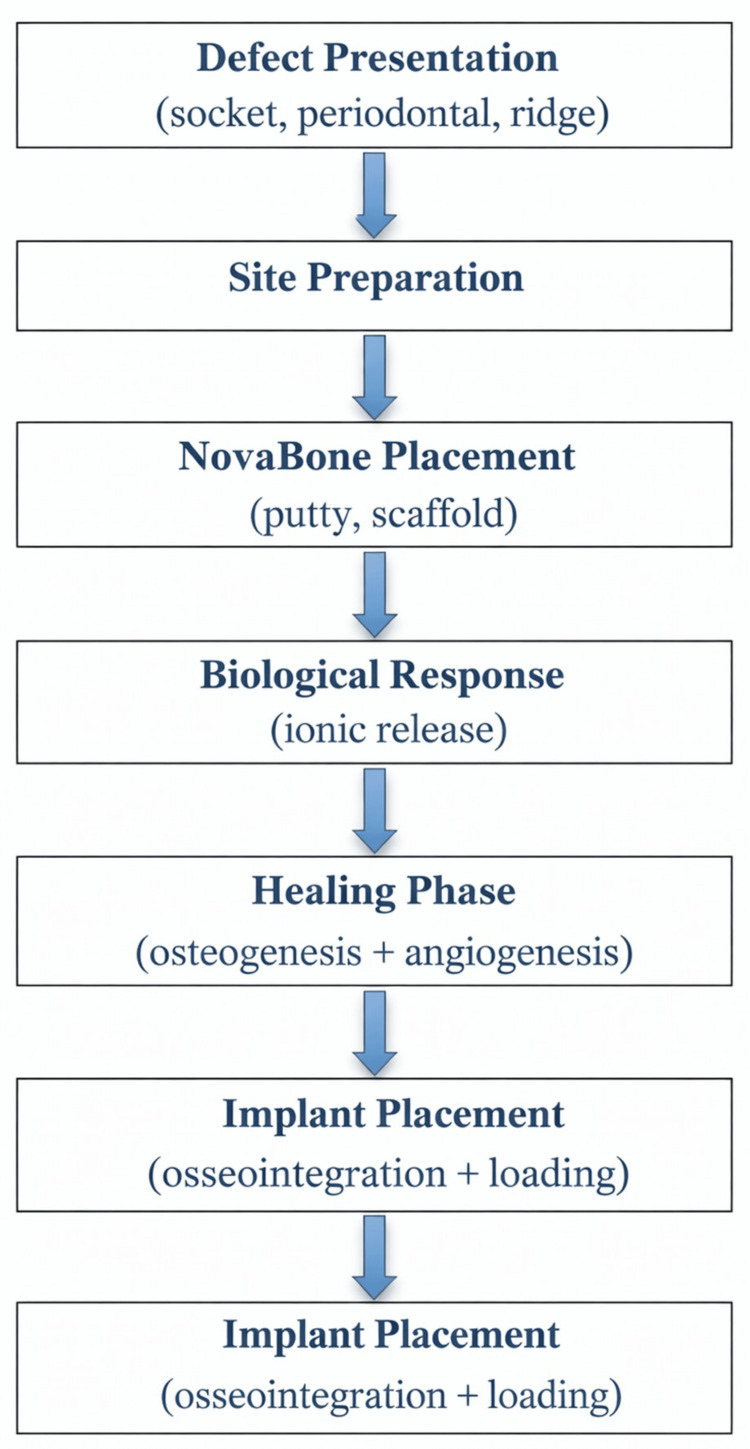
Clinical workflow of NovaBone application in oral bone regeneration from defect presentation to implant-supported rehabilitation Generated by the authors using Microsoft Word (Microsoft Corp., Redmond, WA)

For peri-implant and implant site reconstructive applications, calcium phosphosilicate putty has been investigated as a reconstructive material and has shown clinically useful outcomes in comparative settings (Table 3 and Figure 2) [61].

**Table 3:** Clinical indications and outcomes of NovaBone use

Clinical indication	Main purpose	Reported outcomes	Key references
Socket preservation	Reduce post-extraction ridge collapse	Ridge preservation, new bone formation, and implant site readiness	[47-50]
Ridge augmentation	Improve the deficient ridge contour for implants	Defect fill and implant site development	[51]
Sinus lift procedures	Increase posterior maxillary bone height	Histologic bone formation, graft maturation, and implant placement support	[52-55]
Periodontal intrabony defects	Regenerate periodontal support	Probing pocket depth reduction, clinical attachment level gain, and radiographic bone fill	[56-59]
Furcation defects	Regenerate periodontal tissues in molar furcation lesions	Clinical and radiographic improvement	[60]
Peri-implant defects	Reconstruct the bone around implants	Defect fill and reconstructive surgical success	[61]

Clinical outcomes and evidence synthesis

Bone Regeneration Outcomes

The most consistently reported regenerative outcomes with NovaBone and related calcium phosphosilicate materials are radiographic bone fill and clinical defect resolution. In periodontal defects, clinical and radiographic studies have shown meaningful improvement after NovaBone putty placement [56-60]. In extraction sockets, histomorphometric studies have demonstrated new bone formation after grafting with calcium phosphosilicate putty, indicating that these materials participate in true regenerative healing rather than merely serving as passive fillers [49,50].

Implant-Related Outcomes

Implant-related evidence comes mainly from grafted sockets, sinus augmentation, and reconstructed implant sites. Clinical series have reported successful implant placement in sites treated with calcium phosphosilicate putty [51], and sinus studies have shown that bioactive glass-supported graft maturation can permit implant rehabilitation [52-55]. Evidence specifically reporting implant stability quotient (ISQ) values for NovaBone is limited, but studies using Periotest assessment have suggested acceptable primary stability in implants placed in sockets previously augmented with calcium phosphosilicate putty [62].

Comparative Effectiveness

Comparative studies indicate that NovaBone and related calcium phosphosilicate materials can produce clinically meaningful outcomes similar to other regenerative graft options in selected indications. In peri-implant defects, calcium phosphosilicate putty has been directly compared to bovine bone particulate [61]. In periodontal defects, NovaBone putty has been compared to nanocrystalline hydroxyapatite and older particulate bioactive glass systems, with both test and comparator groups generally showing improvement [56,59]. Current evidence supports NovaBone as a viable synthetic alternative but not as universally superior to autografts or xenografts across all indications [56,59,61].

Safety and Complications

The published clinical literature generally supports the favorable biocompatibility of bioactive glass and calcium phosphosilicate grafts. Major graft-specific adverse effects are not repeatedly highlighted in the available human studies, and most reports describe acceptable healing and clinical tolerance [47,52,56,61]. Still, the existing studies are relatively small and are not primarily designed to detect rare complications, so the most accurate conclusion is that no major recurring safety signal has been identified, rather than that complications are completely absent (Table 4) [47,52,56,61].

**Table 4:** Summary of clinical studies

Study	Design	Indication	Material/comparison	Main finding
Camargo et al. [47]	Clinical study	Extraction socket preservation	Bioactive glass + calcium sulfate	Helped preserve alveolar process dimensions
Kotsakis et al. [49]	Histomorphometric study	Extraction sockets	Calcium phosphosilicate (CPS) putty	New bone formation before implant placement
Mahesh et al. [50]	Histologic study	Socket grafting	CPS putty	Demonstrated regenerative healing in grafted sockets
Babbush et al. [51]	Clinical series	Implant-related grafting indications	CPS putty	Favorable clinical efficacy in implant dentistry
Cordioli et al. [52]	Clinical/histologic study	Maxillary sinus augmentation	Bioactive glass + autogenous bone	Supported sinus graft maturation and implant placement
Menezes et al. [53]	Comparative study	Sinus augmentation	Bioactive glass + autogenous bone	Demonstrated favorable histomorphometric findings
Turunen et al. [55]	Clinical study	Sinus floor augmentation	Bioactive glass granules + autogenous bone	Reduced autogenous bone requirement
Koduru et al. [56]	Comparative clinical study	Intrabony periodontal defects	NovaBone putty versus nanocrystalline hydroxyapatite	Both improved; NovaBone showed regenerative benefit
Sarika et al. [57]	Clinical/radiographic study	Interproximal vertical defects	NovaBone putty with/without platelet-rich fibrin (PRF)	PRF combination showed better outcomes
Agrawal et al. [58]	Clinical study	Periodontal intraosseous defects	CPS putty with/without platelet-rich plasma (PRP)	CPS putty improved outcomes; PRP offered no additive benefit
Gupta et al. [59]	Comparative cone-beam computed tomography (CBCT) study	Grade II furcation defects	NovaBone Dental Putty versus particulate bioactive glass	Putty showed favorable clinical and CBCT outcomes
Biswas et al. [60]	Comparative clinical study	Grade II furcation defects	NovaBone putty versus PRF	Both groups improved clinically
Kotsakis et al. [61]	Randomized controlled trial	Peri-implant osseous defects	CPS putty versus bovine bone	CPS putty was a viable reconstructive option
Mahesh et al. [62]	Comparative implant stability study	Implants in grafted sockets	CPS putty versus naturally healed sockets	Acceptable implant stability by Periotest

Advantages and limitations

NovaBone belongs to the broader class of synthetic bioactive glass grafts, and one of its main clinical advantages is that it avoids the harvesting step required for autogenous bone. This eliminates donor-site morbidity and reduces the added surgical burden associated with secondary graft procurement while still providing a ready-to-use biomaterial for defect filling and regenerative support [63,64]. Because bioactive glass is manufactured rather than harvested, it also offers consistency of supply and avoids some of the biological and acceptance issues associated with human- or animal-derived graft substitutes [63].

A second advantage is its favorable biocompatibility and biological activity. Unlike inert fillers, bioactive glass interacts with surrounding tissues through surface reactions and ionic dissolution, which helps create a microenvironment supportive of osteogenic activity, angiogenesis, and scaffold-guided bone healing [64-66]. In periodontal and oral regenerative literature, bioactive glass materials are commonly described as combining osteoconductive behavior with an osteostimulatory effect mediated by ion release and interfacial apatite formation, which makes them attractive in defects where biological activation is as important as physical space maintenance [64-66].

From a practical standpoint, NovaBone-type putty and injectable concepts also provide handling benefits. Moldable or flowable formulations can adapt to irregular defects, improve retention within contained sites, and potentially reduce the technical difficulties associated with particulate graft placement [67]. These characteristics are clinically relevant in oral surgery, where defects often have complex geometry and where stable graft adaptation can influence early healing [67].

However, the limitations of the current evidence base should be stated clearly. Although many studies report favorable short- and medium-term outcomes, long-term randomized clinical data remain relatively limited, especially for product-specific conclusions in implant-oriented oral regeneration [65,68]. Systematic reviews of periodontal applications also note heterogeneity in surgical technique, defect morphology, adjunctive biologics, comparator materials, and outcome reporting, all of which make direct comparison difficult and reduce the strength of pooled conclusions [65,68]. In addition, treatment cost may influence clinical adoption. Although synthetic grafts avoid donor-site surgery, newer composite, injectable, and digitally fabricated biomaterial systems may increase material and processing costs, which could limit routine use depending on the clinical setting and indication [67,69].

The future direction of NovaBone-related technology is likely to move toward more personalized and defect-specific regenerative systems. Additive manufacturing and scaffold customization now allow the design of patient-specific geometries, controlled porosity, and the spatial distribution of bioactive phases, which may improve adaptation to complex craniofacial defects and permit more predictable regeneration [69,70]. This is particularly relevant for oral and maxillofacial applications, where defect anatomy is often highly individualized and where an ideal graft should combine biological activity with geometric precision [69,70].

Another important direction is integration with growth factors, stem cells, and multifunctional carrier systems. Recent bioactive glass research has increasingly explored composite scaffolds that incorporate osteogenic mediators or cell-compatible matrices to enhance bone formation, angiogenesis, and interface maturation [71,72]. These strategies are promising because they move beyond conventional grafting toward a tissue-engineering model in which the scaffold is not only a defect filler but also a biologically instructive platform [71,72].

Digital and guided regeneration approaches are also likely to become more important. Three-dimensional printing, scaffold simulation, and digitally controlled architecture may help align material degradation, pore structure, and mechanical behavior with the regenerative demands of a given defect [69,70]. In dentistry, these advances could support more precise implant site development, guided augmentation, and personalized regenerative workflows [70].

Despite this progress, a major research priority remains the generation of higher-quality human evidence. Available reviews continue to emphasize the need for better-designed randomized trials with standardized protocols, longer follow-up, and clearer outcome definitions before stronger product-level recommendations can be made for specific oral indications [65,68]. For NovaBone specifically, future studies should aim to distinguish class effects of bioactive glass from formulation-specific benefits in socket preservation, ridge augmentation, sinus grafting, and peri-implant reconstruction [65,68].

In interpreting the available evidence, it is important to distinguish between studies that directly evaluate NovaBone and those that report findings from broader bioactive glass or calcium phosphosilicate materials. NovaBone-specific studies provide the most direct clinical relevance, particularly in periodontal and oral bone regeneration applications. However, where direct NovaBone data are limited, findings from the wider bioactive glass literature may help explain possible biological mechanisms, including ion release, HCA formation, osteogenic stimulation, and bone bonding. These broader findings should be interpreted as supportive rather than definitive evidence for NovaBone-specific clinical performance.

A limitation of this review is that it is narrative in nature and does not include a formal systematic search, risk-of-bias assessment, or quantitative synthesis. In addition, some mechanistic explanations are supported by broader bioactive glass literature rather than NovaBone-specific studies alone. Future well-designed randomized clinical trials with standardized outcome measures, longer follow-up periods, and direct comparison with commonly used graft materials are needed to better define the clinical effectiveness of NovaBone in oral and implant-related bone regeneration.

## Conclusions

The available literature supports NovaBone as a clinically relevant synthetic bioactive graft within contemporary oral regeneration. Its value lies in a combination of biocompatibility, the absence of donor-site morbidity, surface bioactivity, and ion-mediated regenerative signaling that distinguishes it from purely passive scaffold materials. Across oral applications, the strongest direct evidence is in periodontal regeneration, while broader bioactive glass literature also supports its translational relevance to implant site development and reconstructive procedures. From an implant dentistry perspective, NovaBone occupies an important position among modern graft materials because it offers a synthetic alternative that is easier to handle than autogenous grafts and is biologically more active than many conventional inert substitutes. At the same time, current evidence does not justify overstating its superiority across all indications. Its most appropriate interpretation is as a promising mechanism-driven biomaterial whose clinical use should be matched to defect characteristics, treatment goals, and the quality of available evidence. Overall, NovaBone should be viewed not simply as a defect filler but as a bioactive regenerative platform whose clinical potential is best understood through the integration of material science, biological mechanisms, and indication-specific outcomes. Future progress will depend on stronger comparative trials and on the continued development of personalized, composite, and digitally guided regenerative strategies.
